# Nurses’ Roles, Challenges, and Reported Outcomes in Rural and Remote Healthcare: A JBI-Aligned Scoping Review (PRISMA-ScR)

**DOI:** 10.3390/healthcare14101412

**Published:** 2026-05-21

**Authors:** Muteb Aljuhani, Hanadi Dakhilallah, Norah M. Alyahya, Bandar S. Alharbi, Albandari Almutairi, Waleed M. Alshehri, Thurayya Eid, Abdulaziz M. Alodhailah

**Affiliations:** 1 Department of Community Health, Mental and Psychiatric Nursing, Imam Mohammad Ibn Saud Islamic University (IMSIU), Riyadh 11564, Saudi Arabia; 2 Nursing Administration and Education Department, College of Nursing, Imam Mohammad Ibn Saud Islamic University (IMSIU), Riyadh 11564, Saudi Arabia; 3 Community and Psychiatric Mental Health Nursing Department, College of Nursing, King Saud University, Riyadh 11451, Saudi Arabia; 4 Department of Maternal and Child Health Nursing, College of Nursing, King Saud University, Riyadh 11451, Saudi Arabia; 5 Department of Medical-Surgical Nursing, College of Nursing, King Saud University, Riyadh 11451, Saudi Arabia

**Keywords:** rural health, nursing roles, scoping review, outcomes, access to care, telehealth, JBI methodology

## Abstract

Background: Rural and remote health systems are diverse; while many of these settings face persistent workforce shortages and access gaps, not all are underserved. Nurses play a critical role in improving access, continuity, and quality of care in these contexts. However, evidence on their roles, the challenges they face, and the outcomes associated with their contributions remains fragmented. Objective: To map the roles, challenges, and reported outcomes of nurses working in rural and remote healthcare settings, and to examine the quality and scope of the available evidence. Design: This study employed JBI scoping review methodology and is reported in accordance with the Preferred Reporting Items for Systematic Reviews and Meta-Analyses extension for Scoping Reviews (PRISMA-ScR). Methods: Eligible studies involved registered nurses (RNs) and nurse practitioners (NPs) providing care in rural or remote settings and reporting at least one outcome related to patients, services, or health systems. Six bibliographic databases (PubMed/MEDLINE, CINAHL, Embase, Scopus, Web of Science, Cochrane Library) plus Google Scholar for supplementary grey literature retrieval and targeted grey literature were searched (from 1 January 2000 to 30 September 2025). The lead author conducted screening and data extraction, supported by a 10% calibration pilot and structured peer debriefing. Design-specific critical appraisal was undertaken descriptively to inform interpretation but did not determine inclusion. Results: From 22 primary empirical studies (plus 2 contextual-only entries; 24 total, nurses’ roles clustered into direct clinical care, care coordination/navigation, telehealth facilitation, and health promotion. Reported outcomes were predominantly in access/utilization (e.g., time-to-care), quality and safety indicators, and patient-reported outcomes/experiences; clinical endpoints were less common. Conclusions: Nurses in rural and remote settings enact broad, adaptive roles that appear to support healthcare access and service continuity. The evidence base is predominantly descriptive, and causal claims about effectiveness cannot be drawn from the available studies. Standardized outcome frameworks, multi-reviewer methodologies, and effectiveness-focused primary research are needed to advance this field.

## 1. Background

Rural and remote healthcare systems are complex, diverse, and shaped by geography, population distribution, and resource availability. Many such communities face persistent challenges including workforce shortages, limited access to specialist services, and infrastructural constraints. However, these issues are not universal: some rural regions are relatively well resourced, while others experience chronic service delivery gaps [1,2]. Within these varied contexts, nurses frequently serve as the first point of contact for patients, delivering acute and emergency care, chronic disease management, health promotion, care coordination, telehealth facilitation, and community outreach [3,4,5].

Despite this, the existing literature on rural nursing remains fragmented, often focusing narrowly on workforce characteristics or role descriptions with limited attention to how these roles translate into measurable patient, service, or system outcomes [6,7]. Earlier syntheses have addressed workforce retention [6,7], scope of practice [8,9], and nurse-led models of care [10,11], but few have systematically mapped the full range of nursing roles alongside documented outcomes using a structured taxonomy. This review addresses that gap by combining PCC-framed role mapping with a Donabedian-inspired outcome taxonomy, providing a more complete account of what rural nurses do and what their contributions produce in measurable terms. The deliberate focus on RNs and NPs, excluding students, assistants, and unlicensed personnel, ensures conceptual precision and enables meaningful cross-study comparison.

For this review, rural areas are defined as non-metropolitan regions characterized by lower population density and longer travel times to service hubs; remote areas are more sparsely populated and geographically isolated [12,13], following OECD and WHO frameworks. Country-specific national classifications were accepted when transparently reported in primary studies.

To bring structure to the complexity of outcome evidence, the review adopts a Donabedian-inspired taxonomy [14] alongside PROM and PREM frameworks [15,16]. Five outcome categories are considered: patient-reported outcomes (self-efficacy, symptom burden, perceived health status); patient-reported experiences (satisfaction, cultural safety, trust); service utilization and access indicators (time-to-care, ED presentations, referrals); quality and safety measures (guideline adherence, error rates); and clinical or proxy outcomes (disease control, complications, admissions).

The research question is structured using the Population, Concept, and Context (PCC) framework, which is recommended by JBI for scoping reviews and is better suited to mapping heterogeneous evidence across diverse settings than the PICO framework used in effectiveness reviews [17]. The research question is as follows: Among RNs and NPs working in rural and remote healthcare settings, what roles and functions are described, what challenges are reported, and what outcomes at patient, service, and system levels have been documented?

## 2. Methods

### 2.1. Design and Reporting

The authors conducted a JBI-aligned scoping review to map the extent, range, and nature of evidence on RNs and NPs working in rural and remote healthcare, including their roles, challenges, and reported outcomes. The review follows the PRISMA-ScR reporting guideline (checklist provided in Supplementary File S1) [18,19]. An a priori protocol was registered (CRD42024598184); deviations, including the refinement of the outcome taxonomy, are noted below.

### 2.2. Eligibility Criteria (PCC Framework)

Population (P): Licensed RNs and NPs providing care in rural or remote settings. Studies focused exclusively on students, unlicensed personnel, or mixed cadres without separable RN/NP data were excluded.

Concept (C): Descriptions of nursing roles and functions, practice challenges and enablers, and reported outcomes at patient, service, or system levels.

Context (C): Rural and remote healthcare as defined by OECD/WHO guidance. Country-specific classifications were accepted when transparently reported [20,21].

Study designs: Primary empirical research was eligible, including qualitative, quantitative, and mixed-methods designs, observational studies, pragmatic trials, program evaluations, and implementation studies. Systematic reviews, meta-analyses, editorials, commentaries, and protocols were excluded to avoid double counting. This criterion was applied consistently throughout all stages of screening and extraction. Two exceptions were made post hoc and are explicitly flagged [22], a mini-review on dehydration prevention in rural elderly populations, and [23] a case study involving logistics administrators rather than direct nursing participants. Both were retained in Table 1 as ‘contextual only’ boundary cases to provide interpretive framing for specific sub-themes, but neither contributes to the 22-study formal synthesis, and both are clearly labelled throughout.

Time window: 1 January 2000 to 30 September 2025.

Outcomes: At least one extractable outcome from the taxonomy: PROMs, PREMs, access/utilization, quality/safety, or clinical/proxy.

Language: No a priori language restrictions were imposed. Translation was attempted for potentially eligible non-English studies. Studies were not excluded solely on grounds of limited full-text access; interlibrary loan and author contact were pursued where feasible.

### 2.3. Information Sources

The following six bibliographic databases were searched from 1 January 2000 to 30 September 2025: PubMed (incorporating MEDLINE), CINAHL, Embase, Scopus, Web of Science Core Collection, and Cochrane Library. Note: PubMed and MEDLINE (Ovid) appear as separate rows in Table 1 because distinct platform-specific syntax was developed for each interface; however, they index substantially overlapping content and are counted as one source in the database total of six. Google Scholar was searched as a supplementary grey literature source and is listed separately in Table 1 for transparency; it is not counted among the six bibliographic databases. Targeted grey literature sources (WHO, OECD, national ministries of health, rural workforce agencies) were also searched. Reference lists and forward citations of included studies were scanned. A medical librarian peer-reviewed the strategy using PRESS principles [24].

### 2.4. Search Strategy

Search strings were harmonized across databases combining controlled vocabulary (MeSH/Emtree) with keywords and truncation. Core search blocks included:

Nursing block: nurs* (truncation), “registered nurse*”, “nurse practitioner*”, “advanced practice nurs*”.

Setting block: rural, remote, frontier, nonmetropolitan, non-metropolitan, outer regional, isolated community.

Role/model block: “nurse-led”, “care coordination”, “navigation”, “telehealth”, “telemedicine”, “primary health care”, “community health”, “advanced practice”, “continuity”, “community-based”, “outreach”.

Outcome enhancers: “patient-reported outcome*”, “PROM*”, “patient-reported experience*”, “PREM*”, “access”, “utilization”, “quality”, “safety”, “clinical outcome*”.

Where supported, adjacency/proximity operators (e.g., nurs* NEAR/3 rural*) and field restrictions were used to improve precision. Deduplication was performed in EndNote X20 prior to screening. Full reproducible search strings, dates of each run, and limits applied are provided in Supplementary File.

Table 1 presents the database-specific search strings applied across all sources. The strings shown are the core Boolean syntax; full reproducible strings including all field tags, adjacency operators, and MeSH/Emtree vocabulary are provided in Supplementary File. The table strings should be read alongside the Methods description of search block terminology, which specifies the complete set of terms used.

### 2.5. Selection Process

Title/abstract screening was followed by full-text review against eligibility criteria. A calibration phase was applied to approximately 10% of records to refine decision rules, and borderline cases were resolved through structured peer debriefing with two experienced colleagues. Although independent dual screening was not feasible for this review, this limitation is explicitly acknowledged in Section 2.9 and in the Discussion. Reasons for full-text exclusion were documented systematically.

### 2.6. Data Charting

A structured charting form (piloted and iteratively refined) captured study identifiers, design and sample characteristics, nurse cadre and role category, service model, contextual features, outcomes by taxonomy category, measurement instruments, implementation factors, and design-appropriate appraisal ratings.

### 2.7. Critical Appraisal

**Figure 1:**
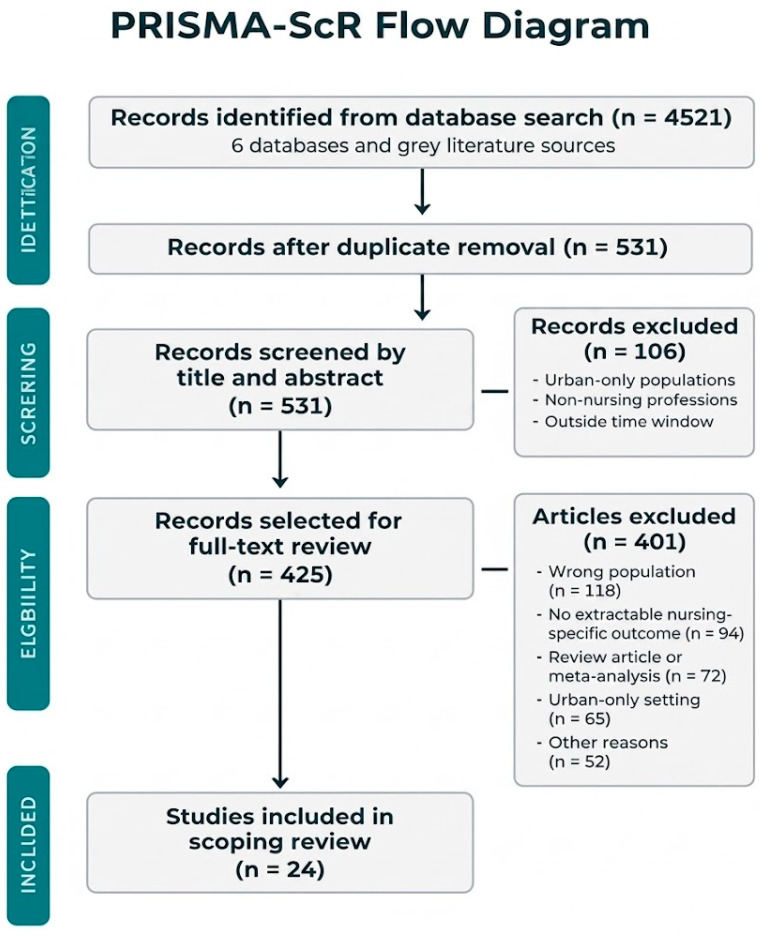
PRISMA flow chart.

Design-specific critical appraisal was undertaken descriptively to aid interpretation and contextualize confidence in findings; it did not determine inclusion [17]. CASP was used for qualitative studies, the Newcastle-Ottawa Scale (or JBI equivalents) for observational designs, and RoB 2 for the randomized trial. For mixed-methods studies, the dominant component was appraised and integration quality noted. Appraisal findings are summarized in the results and tables. Because different tools use different domain labels, Figure 1 presents a harmonized traffic-light summary using three generic cross-design domains: (D1) participant/sample representativeness and selection, (D2) measurement and procedural fidelity, and (D3) outcome reporting completeness. Each included study was mapped to these three domains regardless of the original tool used, with judgments translated as follows: tool-specific domains addressing sampling and recruitment were mapped to D1; domains addressing measurement validity, intervention fidelity, or procedural adherence to D2; and domains addressing outcome completeness, reporting bias, or follow-up to D3. This harmonization approach is consistent with cross-design synthesis in scoping reviews and is explicitly acknowledged as an interpretive simplification rather than a formal risk-of-bias assessment.

### 2.8. Synthesis Aapproach

A descriptive numerical summary (counts by country, design, setting, cadre, role category) was produced alongside a narrative synthesis organized by the outcome taxonomy. Roles and challenges are presented as explanatory context for outcomes. Evidence maps cross-tabulate role categories against outcome families to highlight gaps. No meta-analysis was attempted due to heterogeneity in designs, measures, and contexts.

### 2.9. Limitations of the Review Process

Several methodological limitations require explicit acknowledgment. First, all operational screening, data charting, and appraisal tasks were conducted by the lead author, representing a departure from dual independent reviewer standards. Calibration and peer debriefing were employed but do not fully eliminate selection bias risk. Second, the eligibility criteria were refined between protocol registration and final application, with deviations documented in Supplementary File. Third, the breadth of the charting framework relative to the number of included studies (*n* = 24) increases the risk of uneven extraction. Future reviews should incorporate independent dual screening and a more tightly scoped research question.

## 3. Results

### 3.1. Study Selection and Characteristics

The initial search yielded 4521 records from six bibliographic databases and Google Scholar and grey literature sources. After removing 3990 duplicates, 531 records were screened at the title and abstract level. Of these, 106 were excluded as clearly irrelevant (urban-only populations, non-nursing professions, or outside the time window), leaving 425 records for full-text assessment, as key eligibility criteria (particularly rural/remote classification and nursing-specific outcomes) could not be reliably determined from titles and abstracts alone.

Following full-text review, 401 records were excluded for documented reasons: wrong population (*n* = 118), no extractable nursing-specific outcome (*n* = 94), review article or protocol (*n* = 72), urban-only setting (*n* = 65), and other reasons (*n* = 52). Twenty-two studies met all primary eligibility criteria and are included in the formal synthesis [25,26,27,28,29,30,31,32,33,34,35,36,37,38,39,40,41,42,43,44,45,46], with two additional contextual entries [22,23] retained in Table 2 as boundary-case references but excluded from the synthesis count and the exclusion tally of review articles or protocols (see Table 2 note). The total number of entries in Table 2 is 24, comprising 22 primary empirical studies and 2 contextual-only inclusions. The PRISMA-ScR flow diagram is presented in Figure 1.

### 3.2. Quality Assessment

**Figure 2:**
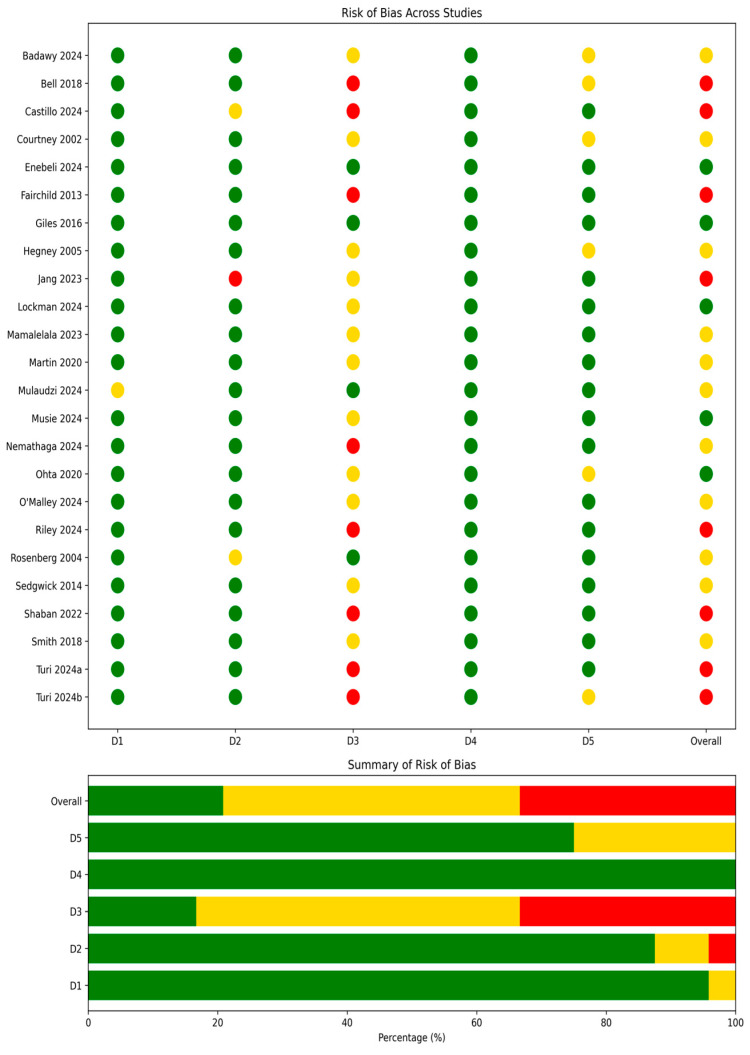
Risk of bias assessment for included studies [25,26,27,28,29,30,31,32,33,34,35,36,37,38,39,40,41,42,43,44,45,46,47,48,49,50,51,52,53,54,55,56,57,58,59,60]. Risk of bias was assessed using a harmonized traffic-light summary chart across the 22 primary empirical studies. Three harmonized domains (D1: participant selection; D2: measurement and procedural fidelity; D3: outcome reporting completeness) were mapped from design-specific appraisal tools (CASP, Newcastle–Ottawa Scale/JBI equivalents, and RoB 2) to enable cross-design comparison. The two contextual-only references [22,23] were excluded from this figure.

Critical appraisal revealed variable methodological quality across the 22 primary empirical studies (the 2 contextual-only entries were not formally appraised). Appraisal domains in Figure 2 are presented using three harmonized cross-design categories (D1: participant selection and representativeness; D2: measurement and procedural fidelity; D3: outcome reporting completeness) to enable visual comparison across qualitative, quantitative, and mixed-methods designs. See Section 2.7 for the full mapping rationale. Most studies demonstrated acceptable practices in participant selection (D1) and concept description (D2). However, notable concerns were identified in outcome measurement and reporting (D3), particularly in studies relying on self-reported or subjective data [35,46]. Several studies showed concerns regarding deviations from intended study procedures (D2) [30,39]. The risk-of-bias figure (Figure 2) reflects a range of judgments; several studies received “some concerns” or high-risk overall ratings.

### 3.3. Main Outcomes

The included studies map nursing roles, challenges, and outcomes across diverse rural and remote healthcare contexts (Table 2). Six themes emerged from the narrative synthesis.

### 3.4. Theme 1: Leadership and Adaptability

Rural nurses consistently demonstrated leadership and adaptability in high-pressure, resource-limited environments. Riley et al. (2024) documented emergent leadership in resuscitation scenarios where equipment and specialist support were scarce [30]. Giles et al. (2016) found that rural nurse consultants focused more on clinical leadership than their urban counterparts [38]. Lockman et al. (2024) showed that nurse navigators’ relationships and beliefs substantially influenced cancer screening uptake and patient trust [42]. Jang et al. (2023) identified prior disaster nursing education and compassion satisfaction as predictors of COVID-19 competency [39]. These studies collectively describe leadership as an emergent feature of rural nursing practice rather than a formally assigned role.

### 3.5. Theme 2: Training and Professional Development

A persistent gap between professional development needs and available opportunities was evident across studies [30]. Fairchild et al. (2013) documented the absence of culturally relevant continuing education in rural facilities [32]. Martin et al. (2020) found that rural midwives reported increased confidence following a structured educational program but continued to face ongoing access barriers [41]. Hegney et al. (2005) reported that rural nurses commonly provided medication education but lacked formal training in interpreter use [27]. The evidence describes the gap consistently but rarely evaluates specific training interventions in comparative designs.

### 3.6. Theme 3: Interprofessional Collaboration

Collaboration challenges were reported across diverse settings. Ohta et al. (2020) identified role vagueness and information flow gaps as primary barriers to effective interprofessional care in rural Japan [28]. Musie and Mulaudzi (2024) found low knowledge levels and negative attitudes toward collaboration with traditional birth attendants, with measurable adverse effects on maternal and neonatal outcomes [36]. Geographic isolation and communication infrastructure deficits compounded these barriers across multiple studies.

### 3.7. Theme 4: Specialized Care Delivery Challenges

Nemathaga et al. (2024) reported inadequate antiepileptic drug supplies and cultural resistance as barriers to epilepsy management in rural South African clinics [37]. Badawy et al. (2024), the only RCT in the review, found that a resilience-building nursing intervention improved psychological wellbeing in older adults, though socioeconomic constraints limited reach [35]. Turi et al. (2024) found that supportive NP work environments were associated with improved substance use disorder care access, with variation across rural settings [40,43]. These studies represent the review’s strongest evidence linking nursing activity to measured outcomes, though the diversity of designs and outcome measures limits aggregation.

### 3.8. Theme 5: Health Promotion and Preventive Roles

Enebeli et al. (2024) documented community health nurses’ central role in health promotion in rural Nigeria despite significant resource constraints [26]. Shaban et al. (2022) reviewed community nursing approaches to dehydration prevention in elderly rural populations [22]. Health promotion was a substantive component of rural nursing across multiple settings, often without dedicated resource allocation. The lack of longitudinal data means sustained impact on preventable disease burden cannot be assessed from this evidence base.

### 3.9. Theme 6: Role Ambiguity and Professional Isolation

Role ambiguity and professional isolation were among the most consistently reported challenges. Bell et al. (2018) described wide variance in role understanding among rural nurse specialists in New Zealand, with unclear expectations creating barriers to effective collaboration [47]. Rosenberg and Canning (2004) identified role diversity and emotional strain as compounding challenges for palliative care nurses working without adequate peer support [29]. Courtney et al. (2002) found that rural nurse executives had broader responsibilities but fewer career advancement opportunities than urban peers [25]. The pattern across these studies suggests that role ambiguity is a structural feature of rural nursing contexts rather than an individually variable experience. Table 3 illustrates the alignment of the identified themes with the study aims.

## 4. Discussion

This scoping review maps the roles, challenges, and reported outcomes of nurses in rural and remote healthcare settings across 22 primary empirical studies from multiple countries and healthcare contexts. The six themes are consistent with the broader rural nursing literature and reflect well-documented structural features of rural practice. The discussion below contextualizes findings, addresses the strength of the evidence base, and identifies where current evidence does and does not support policy prescription.

### 4.1. Leadership and Adaptability

The capacity of rural nurses to lead and adapt in resource-constrained settings is well described across the included literature and aligns with Lee and McDonagh’s [48] account of autonomous decision-making in rural emergencies and Tandan et al.’s [49] analysis of team-based primary care outcomes. However, most evidence is descriptive: leadership capacity is documented rather than evaluated through comparative or experimental designs. This means the claim that leadership translates into measurably better patient outcomes cannot be confirmed from this review alone.

### 4.2. Training and Professional Development

Access to continuing education remains structurally limited for rural nurses, consistent with Smith et al. [50] and Alluhidan et al. [51]. Telehealth and e-learning platforms are consistently identified as viable delivery channels, though included studies do not evaluate specific training modalities through comparative designs. Future research should employ pre-post or controlled designs to determine which training delivery mechanisms produce sustainable competency gains in rural contexts.

### 4.3. Interprofessional Collaboration

Collaboration barriers in rural settings reflect geographic isolation, role vagueness, and communication infrastructure gaps, consistent with Rawlinson et al. [52] and Martin et al. [53]. The evidence points toward system-level solutions, clear protocols, digital infrastructure, and shared decision-making frameworks, but these are more frequently described as needed than evaluated as interventions.

### 4.4. Specialized Care Delivery

Resource gaps in rural specialized care are well established [54,55]. The single RCT in this review (Badawy et al., 2024) [35] provides the clearest evidence of a direct link between nursing intervention and measured outcome (psychological resilience and quality of life). Telehealth is consistently identified as a mitigation strategy for specialist access gaps, but its effectiveness is rarely the direct focus of included studies. The gap between telehealth’s described potential and its evaluated performance represents an important area for future primary research.

### 4.5. Health Promotion

Rural nurses’ health promotion roles align with the public health literature [56,57], and included studies confirm these nurses function as primary disease prevention practitioners in their communities. Resource constraints are the dominant barrier. The absence of longitudinal data means that the sustained population health impact of these activities cannot be quantified from this evidence base.

### 4.6. Role Ambiguity and Professional Isolation

Professional isolation and role ambiguity are associated with burnout and turnover [58,59,60], and the included literature frames these as structural features of rural practice. Targeted interventions, clearer role definitions, mentorship infrastructure, and connected professional networks via telehealth, are recommended in multiple studies but not evaluated in this review’s included studies.

### 4.7. Interpretation of Evidence Strength

A candid assessment of this review’s evidence base is essential. The 22 primary empirical studies in the formal synthesis are predominantly observational, qualitative, or descriptive. Few include comparators, and clinical endpoints are uncommon. The review maps what nurses in rural settings do and what outcomes have been observed; it does not establish causal effectiveness. All instances of language implying established effectiveness have been moderated in this manuscript. Policy recommendations derived from this review should be understood as warranting further effectiveness research rather than as established practice prescriptions.

### 4.8. Limitations

The principal limitations of this review are methodological. Single-reviewer screening and extraction, even when mitigated by calibration and peer debriefing, introduces the possibility of selection bias and inconsistent categorization. The eligibility criteria underwent refinement after protocol registration, with deviations documented. High-income-country dominance of included studies limits transferability to low- and middle-income rural settings. Taken together, confidence in the completeness and reproducibility of the synthesis is moderate. Future reviews on this topic should incorporate independent dual screening and a more tightly scoped research question to strengthen evidentiary credibility.

### 4.9. Implications

Policy: Policymakers should prioritize investment in rural nursing infrastructure, including telehealth, accessible continuing education, and interprofessional collaboration frameworks. These priorities are supported by the described needs across included studies; their effectiveness requires evaluation through rigorous primary research.

Practice: Healthcare institutions in rural areas should support leadership development and provide nurses with the autonomy and resources needed to manage complex cases. Specialized training programs should be tested through comparative designs before wide-scale adoption.

Education: Nursing education programs should incorporate leadership training, health promotion strategies, and rural context preparation. Distance-learning should be expanded to reduce geographic access barriers.

## 5. Conclusions

This scoping review maps the roles, challenges, and reported outcomes of nurses in rural and remote healthcare settings across 22 primary empirical studies (with 2 additional contextual entries in Table 2). Nurses function as first-contact clinicians, care coordinators, telehealth facilitators, and health promotion practitioners, adapting to resource constraints and geographic barriers. The evidence confirms that these roles are substantive and wide-ranging.

The most consistent finding is a gap between the breadth of what rural nurses do and the depth of the outcome measurements that documents like these produce. The evidence base is predominantly descriptive, and the review does not support strong causal claims about nursing effectiveness. Standardized outcome frameworks, investment in longitudinal and comparative study designs, and future reviews incorporating independent dual screening are needed to move this field from role mapping to evidence-based effectiveness evaluation. Workforce planning and policy should be informed by these findings while recognizing their current limitations.

## Figures and Tables

**Table 1:** Search strategy.

Database	Search Terms
PubMed	(“Nurses” [Mesh] OR “Nursing Role” OR “Nursing Intervention*” OR “Nursing Strateg*”) AND (“Healthcare Delivery” OR “Health Services Accessibility” [Mesh] OR “Healthcare Improvement” OR “Quality of Care”) AND (“Rural Health” [Mesh] OR “Rural Setting*”)
MEDLINE (Ovid)	(“Nurses” OR “Nursing Role” OR “Nursing Intervention*” OR “Nursing Strateg*”) AND (“Healthcare Delivery” OR “Healthcare Optimization” OR “Health Services Accessibility”) AND (“Rural Health” OR “Rural Communit*”)
CINAHL	(“Nurses” OR “Nursing Practice” OR “Nursing Role”) AND (“Healthcare Access” OR “Quality of Care” OR “Health Outcome*”) AND (“Rural Area*” OR “Rural Healthcare”)
Embase	(‘nurse’/exp OR ‘nursing role’ OR ‘nursing intervention*’) AND (‘healthcare delivery’ OR ‘health service*’ OR ‘healthcare optimization’) AND (‘rural health’ OR ‘rural area*’)
Scopus	TITLE-ABS-KEY (“Nurses” OR “Nursing Role” OR “Nursing Intervention*”) AND TITLE-ABS-KEY (“Healthcare Delivery” OR “Healthcare Improvement” OR “Health Services Accessibility”) AND TITLE-ABS-KEY(“Rural Setting*” OR “Rural Communit*”)
Web of Science	TS = (“Nurses” OR “Nursing Role” OR “Nursing Intervention*”) AND TS = (“Healthcare Delivery” OR “Healthcare Optimization” OR “Quality of Care”) AND TS = (“Rural Health” OR “Rural Setting*”)
Cochrane Library	(“Nurses” OR “Nursing Role” OR “Nursing Intervention*”) AND (“Healthcare Delivery” OR “Health Services Accessibility”) AND (“Rural Health” OR “Rural Area*”)
Google Scholar	(“Nurses” OR “Nursing Role” OR “Nursing Intervention*” OR “Nursing Strateg*”) AND (“Healthcare Delivery” OR “Healthcare Access” OR “Quality of Care”) AND (“Rural Area*” OR “Rural Healthcare” OR “Rural Communit*”)

Note: An asterisk (*) is used as a truncation symbol to include various word endings (e.g., “Intervention*” includes “Intervention” and “Interventions”).

**Table 2:** Extraction summary of included studies.

Author(s) & Year	Country/Setting	Design	Sample	Nursing Role	Key Outcomes	Limitations
[25] Courtney et al. (2002)	Australia, rural/remote	Cross-sectional quantitative	147 nurse executives	Leadership, role comparison	Fewer development opportunities; broader roles in rural	Response rate
[29] Rosenberg & Canning (2004)	Australia, rural/remote	Mixed-methods	31 nurses	Palliative care	High emotional strain; development needs	Low response rate
[27] Hegney et al. (2005)	Australia, rural/remote	Cross-sectional survey	668 RNs	Patient education, interpreter use	<50% provided adequate education; limited interpreter use	Self-report bias
[32] Fairchild et al. (2013)	USA, rural Midwest	Qualitative	40 facilities	Continuing education	Need for culturally relevant CE; improved skills when provided	Limited geographic scope
[44] Sedgwick et al. (2014)	Canada, rural hospitals	Observational	15 nurses	Clinical reasoning/decision-making	Novice nurses relied on peers; peer support critical for decisions	Small sample; simulation setting
[38] Giles et al. (2016)	Australia, rural	Mixed-methods	Nurse consultants	Clinical leadership	More clinical leadership in rural vs. urban; higher patient contact	One health district
[47] Bell et al. (2018)	New Zealand, rural	Descriptive exploratory	4 interviews	Nurse specialist roles	Wide variance in role clarity; collaboration barriers	Small sample
[46] Smith et al. (2018)	Scotland, rural	Mixed-methods longitudinal	41 nurses	Sensory impairment training	Increased awareness and referral rates post-training	Small sample; limited region
[41] Martin et al. (2020)	Australia, rural midwifery	Retrospective audit	97 midwives	Maternity education program	Increased confidence in maternity care	Retrospective; no causality
[28] Ohta et al. (2020)	Japan, rural	Qualitative	13 homecare nurses	Interprofessional collaboration	Role vagueness and information gaps impair collaboration	Small sample; single region
[22] Shaban et al. (2022) [Mini-review—contextual only]	Saudi Arabia, rural	Mini-review	10 studies	Dehydration prevention in elderly	Nurses promote hydration; face environmental/physiological challenges	Mini-review scope; limited generalizability
[31] Mamalelala et al. (2023)	Botswana, rural health	Qualitative descriptive	26 nurses	Emergency patient transport	Infrastructure and decision-authority gaps impede transport effectiveness	Context-specific
[39] Jang et al. (2023)	South Korea, rural	Cross-sectional	204 nurses	Disaster nursing competencies	Compassion satisfaction and prior training predict competency	Self-reporting bias
[30] Riley et al. (2024)	Australia, rural	Ethnographic	2 hospitals	Resuscitation care leadership	Adaptability and leadership under resource constraints	2 sites only
[23] Castillo et al. (2024) [*Logistics administrators—contextual only*]	Spain, rural	Case study	Logistics administrators	Home care routing logistics	Routing algorithms improved service access in depopulated areas	Single province; not direct nursing
[35] Badawy et al. (2024)	Saudi Arabia, rural	RCT	84 older adults	Resilience-building intervention	Improved psychological resilience and quality of life	Short follow-up; limited generalizability
[36] Musie & Mulaudzi (2024)	South Africa, rural	Descriptive cross-sectional	304 midwives	Collaboration with traditional birth attendants	Low knowledge; negative attitudes toward collaboration affected maternal/neonatal outcomes	Self-report; single region
[37] Nemathaga et al. (2024)	South Africa, rural	Qualitative descriptive	20 nurses	Epilepsy management	Drug supply gaps and cultural resistance as primary barriers	Small sample
[40] Turi et al. (2024) [a]	USA, rural	Cross-sectional quantitative	1152 patients	NP work environment and SUD care access	Supportive work environments associated with improved SUD care access	Cross-sectional; no longitudinal follow-up
[43] Turi et al. (2024) [b]	USA, rural	Cross-sectional	126 practices	NP environment and ED utilization for older adults with SUD	Supportive NP environments associated with reduced ED use	Self-reporting biases
[42] Lockman et al. (2024)	USA, rural cancer care	Qualitative	PCPs and nurse navigators	Cancer care coordination	Trust barriers and fragmented systems limited screening effectiveness	One health system only
[26] Enebeli et al. (2024)	Nigeria, rural	Qualitative	10 community health nurses	Health promotion in primary care	Nurses were key promoters despite resource constraints	Small sample; narrow region
[34] O’Malley et al. (2024)	Australia, rural emergency	Qualitative	Rural ED nurses	Paediatric emergency care	Fear and anxiety from insufficient paediatric training	Single setting

Note: ‘Contextual only’ entries [22,23] are retained as boundary-case inclusions with explicit interpretive caveats. See Supplementary File for full extraction data. Ref [22] is a mini-review that does not meet the primary empirical study criterion stated in the eligibility criteria; it is retained only to provide contextual framing for dehydration prevention in rural elderly populations and is explicitly excluded from the formal synthesis and study count. Ref [23] reports logistics administrators rather than direct nursing participants; it is similarly retained as contextual background for home care routing in depopulated rural areas and excluded from the formal synthesis and count. Neither study contributes to the 24-study synthesis total. The formal included sample therefore consists of 22 primary empirical studies plus these two contextual entries, which together are referenced in narrative passages as descriptive context only.

**Table 3:** Alignment of themes with study aims.

Theme	Roles	Challenges	Outcomes
Leadership and Adaptability	√	√	√
Training and Professional Development	√	√	Indirect *
Interprofessional Collaboration	√	√	√
Specialized Care Delivery	√	√	√
Health Promotion and Prevention	√	√	√
Role Ambiguity and Isolation	√	√	Indirect *

* Indirect: The theme is structurally linked to outcomes (e.g., training gaps affect care quality) but included studies did not directly measure outcome effects attributable to training or role ambiguity through controlled designs.

## Data Availability

All data generated or analyzed during this study are included in this published article and its Supplementary Information Files.

## Supplementary Materials

The following supporting information can be downloaded at: https://www.mdpi.com/article/10.3390/healthcare14101412/s1, File S1: PRISMA 2020 Checklist.
